# Central venous catheter-related nosocomial bloodstream infections in children on long-term parenteral nutrition: the impact of the move to a new university hospital

**DOI:** 10.1186/2047-2994-4-S1-P206

**Published:** 2015-06-16

**Authors:** M-L Valdeyron, N Peretti, J Grando, P Vanhems

**Affiliations:** 1 Infection Control and Prevention, Hospices Civils de Lyon - Groupement Hospitalier Est, Bron, France; 2 Pediatric Hepato-gastroenterology and Nutrition Department, Hospices Civils de Lyon - Groupement Hospitalier Est, Bron, France; 3 Infection Control and Prevention, Hospices Civils de Lyon, Lyon, France

## Introduction

Insufficient training in CVC management contributes in the development of CRBSIs

## Objectives

This study evaluated the impact of moving to a new university paediatric hospital on the incidence of central catheter-related blood stream infections (CRBSIs) among children on long-term parenteral nutrition.

## Methods

This retrospective study covered from April 2007 to March 2014, starting a year prior to move the children to a new hospital in April 2008, and continuing for 6 years following the move. During this observational period, data from all children hospitalized in a hepato-gastroenterology and nutrition unit of a paediatric tertiary hospital who received parenteral nutrition (PN) for more than 15 days were analysed.

## Results

During this 7-year study, 183 children aged 4.6 ± 0.5 years received prolonged PN. Intestinal diseases were the main aetiologies (89%), primarily short bowel syndrome (18.4%), Hirschsprung disease and Chronic Intestinal Pseudo-Obstruction syndrome (CIPO) (13.5%) and inflammatory bowel disease (13.8%). The mean durations of hospitalization and of PN during hospital stay were, respectively, 70±2.1 and 55.7±3.6 days. During the study period, 151 CRBSIs occurred in 77 children (the attack rate was 42% of all patients), i.e. 14.8 septic episodes per1000 PN-days and 12.0 septic episodes per1000 CVC-days. No patient died of a central venous catheter-related infection.

However, following the move from the older hospital to the newer one, the rate of CRBSIs significantly doubled, from 3.9 to 8.8 per 1000 CVC-days (p=0.02). During the following 4 years, the incidence of CRBSIs tended to increase between the 2^nd^ and the 5^th^ year after the move: 11.3 (p=0.5); 21.4 (p=0.01); 17.3 (p=0.4), 20.3 per 1000 (p=0.6) CVC-days. After evaluations by the Department of Infection Control, nurse training and stabilization of the nursing team, the incidence decreased significantly from 20.3 to 11.1/1000 CVC-days during the 6^th^ year after the move (p=0.01).

## Conclusion

Our results revealed the deleterious impact of the hospital move on the CRBSI incidence rate in hospitalized children on PN, and the necessity in having a trained, experienced and stable team of nurses to prevent nosocomial infections.

## Disclosure of interest

None declared.

